# Increased mRNA Levels of Sphingosine Kinases and S1P Lyase and Reduced Levels of S1P Were Observed in Hepatocellular Carcinoma in Association with Poorer Differentiation and Earlier Recurrence

**DOI:** 10.1371/journal.pone.0149462

**Published:** 2016-02-17

**Authors:** Baasanjav Uranbileg, Hitoshi Ikeda, Makoto Kurano, Kenichiro Enooku, Masaya Sato, Daisuke Saigusa, Junken Aoki, Takeaki Ishizawa, Kiyoshi Hasegawa, Norihiro Kokudo, Yutaka Yatomi

**Affiliations:** 1 Department of Clinical Laboratory Medicine, The University of Tokyo, Tokyo, Japan; 2 Hepato-Biliary-Pancreatic Surgery Division, Department of Surgery, The University of Tokyo, Tokyo, Japan; 3 Department of Integrative Genomics, Tohoku Medical Megabank Organization, Miyagi, Japan; 4 Graduate School of Pharmaceutical Sciences, Tohoku University, Miyagi, Japan; 5 CREST, JST, Japan; Medical University of South Carolina, UNITED STATES

## Abstract

Although sphingosine 1-phosphate (S1P) has been reported to play an important role in cancer pathophysiology, little is known about S1P and hepatocellular carcinoma (HCC). To clarify the relationship between S1P and HCC, 77 patients with HCC who underwent surgical treatment were consecutively enrolled in this study. In addition, S1P and its metabolites were quantitated by LC-MS/MS. The mRNA levels of sphingosine kinases (SKs), which phosphorylate sphingosine to generate S1P, were increased in HCC tissues compared with adjacent non-HCC tissues. Higher mRNA levels of SKs in HCC were associated with poorer differentiation and microvascular invasion, whereas a higher level of SK2 mRNA was a risk factor for intra- and extra-hepatic recurrence. S1P levels, however, were unexpectedly reduced in HCC compared with non-HCC tissues, and increased mRNA levels of S1P lyase (SPL), which degrades S1P, were observed in HCC compared with non-HCC tissues. Higher SPL mRNA levels in HCC were associated with poorer differentiation. Finally, in HCC cell lines, inhibition of the expression of SKs or SPL by siRNA led to reduced proliferation, invasion and migration, whereas overexpression of SKs or SPL enhanced proliferation. In conclusion, increased SK and SPL mRNA expression along with reduced S1P levels were more commonly observed in HCC tissues compared with adjacent non-HCC tissues and were associated with poor differentiation and early recurrence. SPL as well as SKs may be therapeutic targets for HCC treatment.

## Introduction

Sphingosine 1-phosphate (S1P) is a bioactive lipid mediator that functions in a wide variety of cellular responses. S1P was first shown to play a role as an intracellular messenger in the mitogenic activity of PDGF or serum in cultured fibroblasts [1]. Additionally, intracellular levels of S1P and its precursor ceramide have been demonstrated to determine cell survival or death [2]. In contrast, some of the diverse effects of S1P, such as stimulation of cell proliferation or contractility, have been shown to be sensitive to pertussis toxin [3] or ADP-ribosyltransferase C3 from *Clostridium botulinum* [4]. These findings indicate that S1P, as an extracellular mediator, activates a receptor coupled to G protein(s). In fact, S1P acts via at least five high-affinity G protein-coupled receptors referred to as S1P_1–5_ [5]. Furthermore, substantial evidence for the phenotypes of S1P receptor mutants [6–9] suggests that S1P has normal roles *in vivo* as well as potentially pathophysiological roles as a circulating paracrine mediator that is stored and released from platelets [10] or erythrocytes [11].

**Fig 1:**
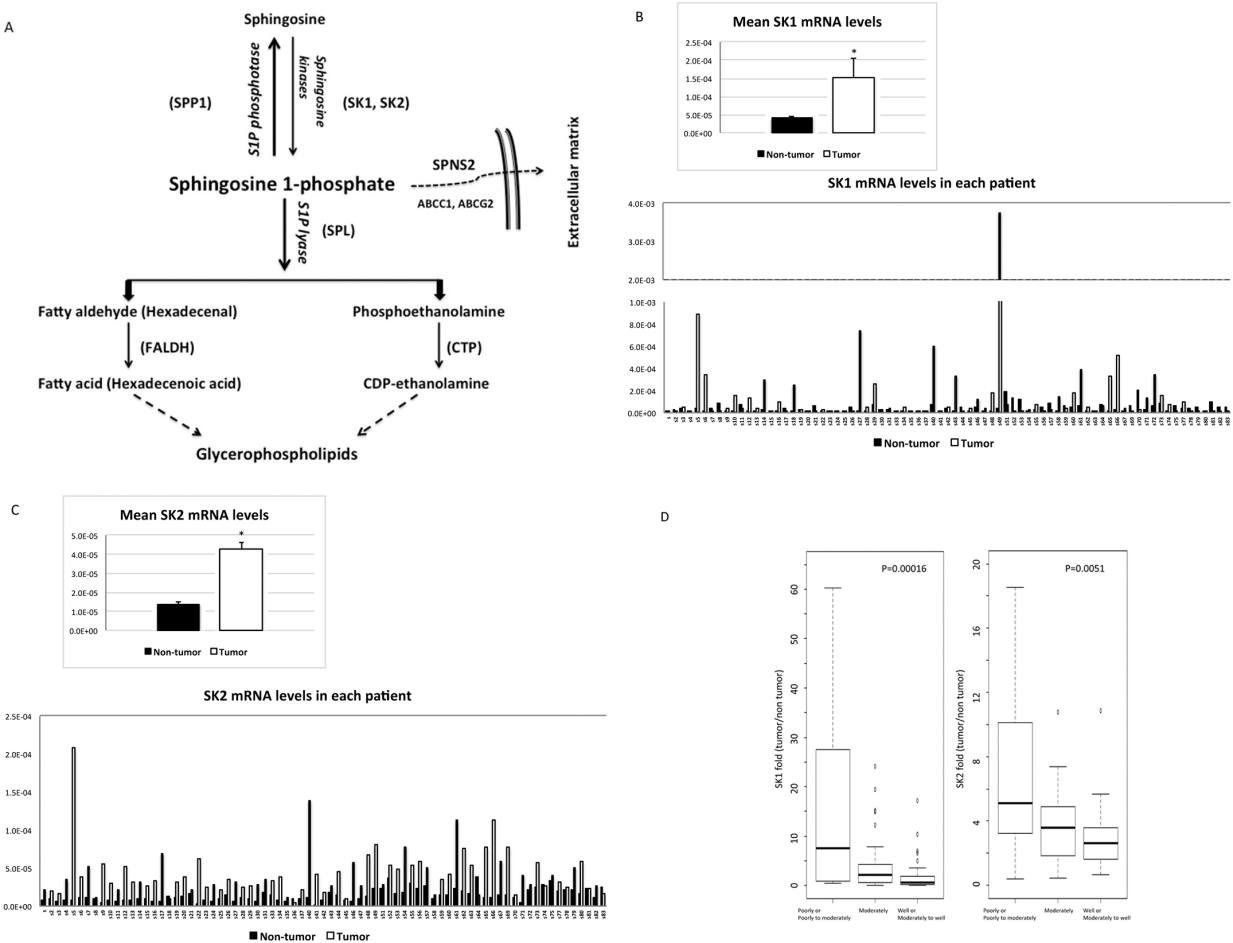
Enhanced SK1 and SK2 mRNA expression in HCC tissues and its association with poorer differentiation. (A) The metabolic pathways involved in the formation and degradation of S1P are depicted. SK1 (B) and SK2 (C) mRNA levels were increased in HCC compared with adjacent non-tumorous tissues in 54.5% and 93.5% of the patients, respectively; the mean mRNA expression level of SK1 and SK2 in HCC tissues was 3.8-fold and 3.0-fold higher, respectively, than that in non-tumorous tissues (*P* = 0.02 and *P* <0.0001, n = 77). (D) The mRNA expression levels of both SKs in HCC tissues compared with those in non-tumorous tissues correlated with the degree of tumor differentiation.

Recent accumulating evidence indicates that S1P also plays an important role in the pathophysiology of cancer [12]. S1P activates nuclear factor kappa B and signal transducer and activator of transcription 3 inflammatory pathways, linking this lipid to colitis-associated cancer [13]. S1P is required for vascular development, as indicated by evidence showing that S1P receptor-null murine embryos display defects in vascular maturation [14] and that S1P plays a role in tumor angiogenesis [15]. Moreover, as mentioned above, intracellular S1P levels are assumed to be determinants of cell survival or death [2], which supports a role for S1P in the biology of cancer. S1P is generated from sphingosine through the actions of sphingosine kinase (SK) enzymes (Fig 1A). There are two isoforms of SK (SK1 and SK2), which differ in terms of their tissue distribution. Because of the potentially close association between S1P and cancer, SKs have been extensively examined, and increased SK1 mRNA and/or protein expression has been reported in cancers of the stomach [16], lung [16], brain [17], colon [16], and kidney [16], as well as in non-Hodgkin lymphoma [18] and breast cancer [16].

In contrast, we focused on a potential role for S1P in the pathophysiology of the liver. In the course of experiments that were conducted to clarify the relationship between S1P and the pathophysiology of the liver, we have shown that S1P has an inhibitory effect on hepatocyte proliferation [9,19]. In contrast, S1P has a stimulatory effect on the proliferation and contraction of hepatic stellate cells [20]. In agreement with these findings, S1P has been shown to play a stimulatory role in hepatic fibrosis [9], in which it enhances portal vein pressure [21]. Furthermore, an S1P receptor 2 antagonist effectively reduces portal vein pressure in rodents with portal hypertension [22]. Other evidence further indicates that S1P plays a key role in wound healing [23] and fibrosis [24–26] in the liver. Collectively, S1P is assumed to play an important role in the pathophysiology of liver fibrosis. However, little is known about S1P and hepatocellular carcinoma (HCC), which often develops in individuals with advanced fibrosis in the liver [27]. In the current study, we sought to examine a potential role for S1P in the pathophysiology of HCC.

## Patients and Methods

### Subjects

Patients with HCC who were treated at the Hepatobiliary Pancreatic Surgery Division, Department of Surgery, at the University of Tokyo Hospital between January 2013 and October 2014, provided consent to be enrolled in this study. Sufficient HCC tissues and adjacent non-tumorous tissues for the analysis of SK mRNA expression were obtained from 77 patients. All of the enrolled patients underwent liver resection, among whom 47 developed primary HCC and 30 developed recurrent HCC.

This study was conducted in accordance with the ethical guidelines of the 1975 Declaration of Helsinki and was approved by the Institutional Research Ethics Committee of the authors’ institution. Written informed consent was obtained from the patients for the use of clinical samples.

### Measurement of Ceramide, Sphingosine and S1P

The samples were prepared as previously reported [28]. Approximately 10–50 mg of liver tissue was placed in a sample tube (2.0 mL) and snap-frozen in liquid nitrogen. Two-hundred microliters of formic acid (0.1% (volume fraction) in methanol) containing the internal standard solution (10.0 ng/mL, mixture of sphingosine (Sph) 17:1, S1P 17:1 and ceramide (Cer) d18:1/17:0) was added to the frozen sample. The mixture was then homogenized using a lysis and homogenization system (Precellys) (5,000 × rpm, 15 sec, 1 Zr bead). The samples were centrifuged at 16,400 × g for 20 min at 4°C, and the supernatants were passed through a 96-well plate for deproteinization (Sirocco, Waters). Finally, 5-μL aliquots of the deproteinized samples were analyzed by LC-MS/MS. The LC-MS/MS system consisted of a NANOSPACE SI-II HPLC (Shiseido) and a TSQ Quantum Ultra triple quadrupole MS (Thermo Fisher Scientific) equipped with a heated-electrospray ionization-II (HESI-II) source. Sphingolipids were analyzed by LC-MS/MS in the selected reaction monitoring (SRM) mode, and the optimal conditions are provided in S1 Table. The ratio of the peak area of the analyte to the internal standard was used to quantify the calibration curves (0.05–100 ng/mL) for each standard solution.

### Quantitative Real-Time PCR of Enzymes Involved in S1P Metabolism

Quantitative real-time PCR was performed as previously described [29]. The primer pairs used were as follows: human SK1: 5’-CTGGCAGCTTCCTTGAACCAT-3’ and 5’-TGTGCAGAGACAGCAGGTTCA-3’; human SK2: 5’-CCAGTGTTGGAGAGCTGAAGGT-3’ and 5’-GTCCATTCATCTGCTGGTCCTC-3’; human S1P lyase (SPL): 5’-GCCAGAGAGTTTATGGTCAAGGTT-3’ and 5’-CAACTTGTCTTGAATCTTACGACCAA-3’; human S1P phosphatase 1 (SPP1): 5’-GCCGCTGGCAGTACCCT-3’ and 5’-AATAGAGTGCATTCCCATGTAAATTCT-3’; internal control 18S ribosomal: 5’-GTAACCCGTTGAACCCCATT-3’ and 5’-CCATCCAATCGGTAGTAGCG-3’. Human fatty aldehyde dehydrogenase (FALDH) and phosphoethanolamine cytidyltransferase (CTP) primers and probes (TaqMan Gene Expression Assays) were obtained from Applied Biosystems (Hs00166066_m1 and Hs00161098_m1). The reaction conditions were as follows: incubation for 10 min at 95°C, followed by 40 cycles at 95°C for 15 sec and 60°C for 1 min. The target gene mRNA expression level was quantified relative to 18S ribosomal RNA using the 2^-ΔΔCt^ method (Applied Biosystems, User Bulletin No 2).

### Cells and Cell Culture

The HCC cell line PLC/PRF/5 was obtained from RIKEN BioResource Center (Tsukuba, Ibaraki, Japan), and HuH7 cells were obtained from the Health Science Research Resources Bank, Japan Health Science Foundation. Because these cells had been passaged in our laboratory for more than 6 months after receipt, the cell lines were tested and authenticated using the Short Tandem Repeat method at the Cell Bank of Japan (March, 2015). PLC/PRF/5 cells were maintained in RPMI-1640 supplemented with 10% fetal bovine serum, and HuH7 cells were cultured in DMEM supplemented with 10% fetal bovine serum. All cells were incubated at 37°C with 5% CO_2_. All experiments were performed using cells that were harvested and passaged fewer than 20 times.

### SK1, SK2 and SPL siRNA Transfection

For the RNA interference assays, SK1, SK2 and SPL ON-TARGET plus SMART pool siRNAs (Dharmacon, Chicago, IL) or a negative control siRNA were delivered using DharmaFECT transfection reagent according to the manufacturer’s instructions. The control, SK1 siRNA and SK2 siRNA were used at a concentration of 25 nM for the RNA interference assays to silence SK1 and SK2 mRNA expression. For SPL, the control and SPL siRNAs were used at a concentration of 50 nM. Three independent transfections were performed for each experiment, and all samples were analyzed in triplicate for each transfection.

### Cell Proliferation, Migration and Invasion Assay

Cell proliferation in the HCC cell lines was measured 72 hours after transfection with SK1, SK2, SPL or negative control siRNAs by evaluating BrdU incorporation using the Cell Proliferation ELISA BrdU colorimetric assay (Roche Applied Science, Upper Bavaria, Germany). For SPL-transfected cells, both serum-free and glucose-free culture medium were used to evaluate cell proliferation.

The cell migration and invasion assays were performed according to the manufacturer’s instructions (BD, NJ, USA), as previously described [29].

### SK1, SK2 and SPL Transfection

Cells expressing human SK1, SK2 and SPL protein were constructed using the mammalian cell expression vector p3FLAG CMV-10 containing the corresponding cDNA derived from normal human liver RNA. The primers used for cloning were SK1: 5’- CCCAAGCTTCACCATGGATCCAGCGG -3’ and 5’- ATAAGAATGCGGCCGCTCATAAGGGCTCTTCTG -3’; SK2: 5’- ATAAGAATGCGGCCGCCACCATGAATGGACACCTTGAAGCAG -3’ and 5’- CGGGATCCTCAGGGCTCCCGCCCCG -3’; SPL: 5’- ATAAGAATGCGGCCGCTAAACTATATGCCTAGCACAGA -3’ and 5’- CGGAATTCT CAGTGGGGTTTTGGAGAACCATTC-3’. These primers were designed based on human SK1, SK2 and SPL reference sequences (NM_001142601.1, NM_001204158.2, and NM_003901, respectively). SK1, SK2 and SPL cDNAs were generated by PCR and verified by DNA sequencing.

### Statistical Analysis

All tests for significance were two-tailed, and *P* <0.05 was considered statistically significant. The cumulative incidence of intra- and extra-hepatic recurrence was calculated using the Kaplan-Meier method, and differences among groups were assessed using the log-rank test. A paired t-test was used to analyze differences in the mRNAs levels of enzymes related with S1P metabolism, and the levels of S1P, ceramide, and sphingosine in tumors and corresponding non-tumorous tissues. Data processing and analysis were performed using SPSS software version 17.0 or 19.0 (SPSS, Inc, Chicago, IL).

The results of the *in vitro* experiments are expressed as the means and standard error of the mean (SEM). The results were considered significant when *P*-values were < 0.05.

## Results

### Patient Characteristics

A total of 77 patients with HCC who underwent surgical treatment were analyzed, among whom 47 were diagnosed with primary HCC. It is well known that HCC recurrence is frequently ectopic because the underlying chronic liver disease continues to increase the patient’s risk for hepatocarcinogenesis [30]. Thus, 30 patients with recurrent HCC after curative surgery were analyzed along with the patients with primary HCC. The clinical and laboratory characteristics of the patients are shown in Table 1.

**Table 1:** Patient characteristics.

Parameter	n = 77
Female/Male	17 / 60
Age (years)	69.8 (64.7–75.0)
BMI (kg/m^2^)	22.8 (21.1–25.1)
Types of hepatitis	
1) Hepatitis B (%)	16 (20.8)
2) Hepatitis C (%)	30 (34.0)
3) Alcoholic (%)	11 (14.3)
4) Others (%)	20 (26.0)
Patients with primary HCC/recurrent HCC	47 / 30
Maximum tumor diameter (cm)	2.5 (1.7–5.0)
Number of tumors	
1) Single (%)	49 (63.6)
2) More than 2 (%)	28 (36.4)
White blood cell count (×10^3^/μL)	5.20 (4.40–6.00)
Hemoglobin content (g/dL)	13.5 (12.2–14.3)
Platelet count (×10^4^/μL)	15.9 (12.4–18.3)
CRP (mg/dL)	0.07 (0.03–0.16)
Albumin (g/dL)	4.0 (3.7–4.2)
AST (U/L)	32 (25–51)
ALT (U/L)	28 (19–44)
GGT (U/L)	47 (30–95)
Total bilirubin (mg/dL)	0.7 (0.6–1.0)
Creatinine (mg/dL)	0.82 (0.65–0.91)
Triglyceride (mg/dL)	99 (77–139)
Total cholesterol (mg/dL)	173 (149.5–195.8)
Fasting blood glucose (mg/dL)	101 (93–115)
HbA1c (NGSP) (%)	5.9 (5.6–6.7)
PT-INR	0.96 (0.93–1.01)
ICGR15 (%)	11.6 (8.2–15.4)
AFP (ng/mL)	8.2 (4.1–52.4)
AFP-L3 (%)	2.4 (0.5–15.7)
DCP (mAu/mL)	28 (16–179.5)
Background liver	
1) Fibrosis stage 0 / 1 / 2 / 3 / 4	2 / 16 / 15 / 21 / 23
2) Activity grade 0 / 1 / 2	9 / 50 / 18
Tumor differentiation	
1) Well (%)	8 (10.4)
2) Well to moderate (%)	22 (28.6)
3) Moderate (%)	34 (44.2)
4) Moderate to poor (%)	10 (13.0)
5) Poor (%)	3 (3.9)
Microvascular invasion (+)/(–)	18 / 59

Values are presented as N (%) or the median (P25, P75).

### Enhanced mRNA Expression of SK1 and SK2 in HCC Tissues Compared with Non-Tumorous Tissues and Its Association with Poor Differentiation and Microvascular Invasion

In liver of non-tumorous tissues, the mRNA levels of SK1 were 2.9 times more than those of SK2 (data not shown). As shown in Fig 1B, the mRNA levels of SK1 were increased in HCC tissues from 42 of 77 patients (54.5%) compared to the paired non-tumorous tissues. The mean mRNA expression level of SK1 was 3.8-fold higher in HCC tissues than in non-tumorous tissues, as depicted in Fig 1B. According to the paired t-test, the mRNA expression levels of SK1 were higher in HCC tissues than in non-tumorous tissues. In contrast, Fig 1C shows that increased mRNA levels of SK2 in HCC tissues compared with non-tumorous tissues were observed in almost all patients (93.5%) and that the mean SK2 mRNA expression level was 3.0-fold higher in HCC tissues than in non-tumorous tissues.

Next, we examined the feature(s) of HCC that is associated with high SK1 and/or SK2 mRNA expression levels. Notably, the mRNA expression levels of both SKs in HCC tissues compared with those in non-tumorous tissues correlated with the degree of tumor differentiation. Higher mRNA levels of both SKs in HCC tissues compared with non-tumorous tissues correlated with a poorer degree of tumor differentiation (Fig 1D). Furthermore, the ratio of SK mRNA levels in HCC tissues to those in non-tumorous tissues was higher in HCC tissues with versus without microvascular invasion (Table 2).

**Table 2:** Correlation of the mRNA levels of SKs and SPL with microvascular invasion in HCC tissues (n = 77).

Parameter	Microvascular invasion		*P* value
	(-)	(+)	
SK2 fold (tumor/non-tumor)	2.81 (1.64–4.12)	5.43 (2.91–16.9)	0.026
SK1 fold (tumor/non-tumor)	0.89 (0.32–3.64)	6.67 (1.61–11.8)	0.0096
SPL fold (tumor/non-tumor)	1.47 (0.92–2.46)	1.69 (0.93–2.47)	0.81

Values are presented as the median (P25, P75)

### Reduced S1P Levels in HCC Tissues Compared with Non-Tumorous Tissues

**Fig 2:**
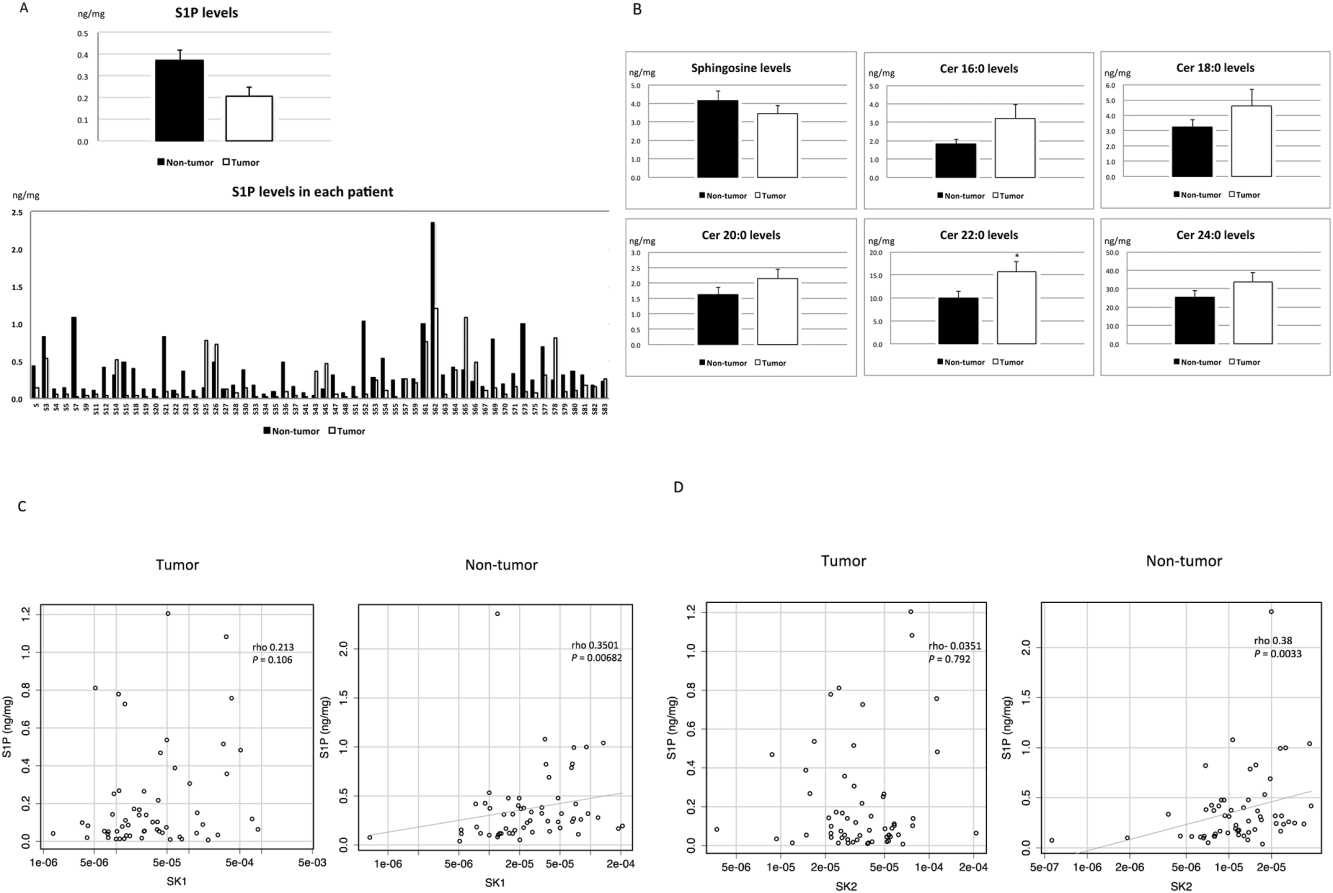
Levels of S1P and its metabolites in HCC tissues and the correlation between S1P levels and SKs. S1P levels (A) were reduced in HCC tissues compared with non-tumorous tissues in 84.5% of the patients; the mean S1P level was lower in HCC tissues than in non-tumorous tissues (*P* = 0.009, n = 58). The levels of sphingosine (Sph) and ceramide (Cer; 16:0, 18:0, 20:0, 22:0 and 24:0) (B) did not differ between HCC tissues and non-tumorous tissues. The asterisk indicates a significant difference. A correlation between S1P levels and SK1 or SK2 (C, D) mRNA levels was not observed in HCC tissues but was observed in non-tumorous tissues (*P* = 0.0068 and 0.0033, n = 58).

We then measured the levels of S1P and its metabolites in HCC and adjacent non-tumorous tissues. Although the mRNA levels of SKs, which are key enzymes in the production of S1P, were highly expressed in HCC tissues compared with adjacent non-tumorous tissues, the levels of S1P were relatively low in HCC tissues compared with non-tumorous tissues, as shown in Fig 2A. Increased levels of S1P were detected in HCC tissues compared with non-tumorous tissues in only 9 of 58 patients (15.5%), and the mean S1P level was lower in HCC tissues compared with non-tumorous tissues. A paired t-test demonstrated that levels of S1P were lower in HCC tissues than in non-tumorous tissues. In contrast, the levels of ceramide (Cer; 16:0, 18:0, 20:0, 22:0 and 24:0) and sphingosine (Sph) did not differ essentially between HCC tissues and non-tumorous tissues, as demonstrated in Fig 2B. Regarding the relationship between the mRNA levels of SKs and the levels of S1P, a correlation between S1P levels and SK1 or SK2 mRNA levels was not observed in HCC tissues but was apparent in non-tumorous tissues (Fig 2C and 2D). These results suggest that S1P metabolism in HCC tissues may be distinct from that in non-tumorous tissues.

### Activation of the SPL Pathway in HCC Tissues Compared with Non-Tumorous Tissues

**Fig 3:**
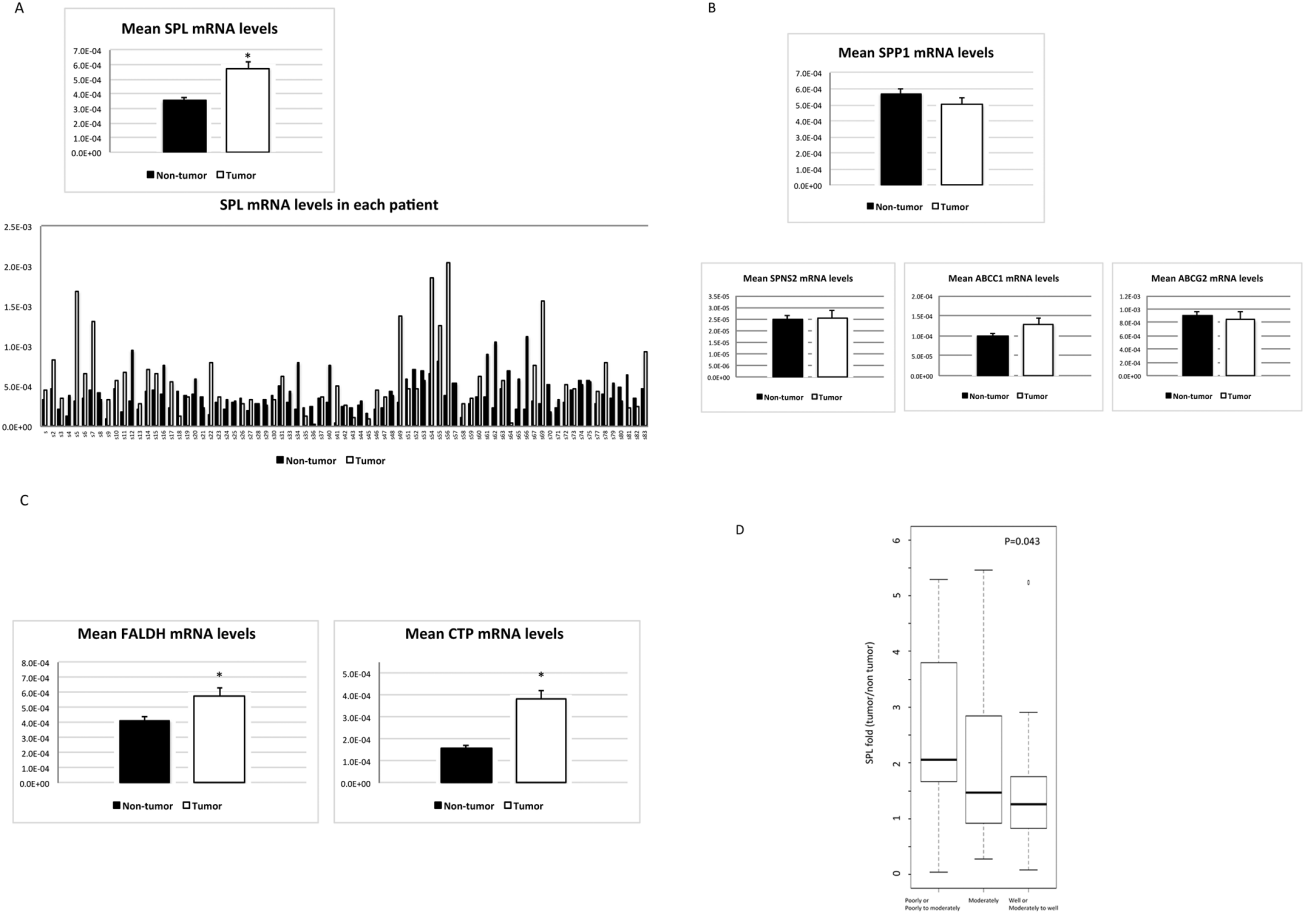
SPL pathway activation in HCC. (A) As an enzyme that degrades S1P, SPL mRNA levels were elevated in HCC tissues compared with non-tumorous tissues in 70.1% of the patients; the mean SPL mRNA level was higher in HCC tissues than in non-tumorous tissues (*P* < 0.0001, n = 77), but SPP1 mRNA levels did not differ between HCC and non-tumorous tissues (B). In contrast, the mRNA levels of S1P transporter, SPNS2, ABCC1 and ABCG2 (B) were not altered in HCC tissues compared with non-tumorous tissues. The mRNA levels of FALDH (C), which catalyzes the formation of hexadecenoic acid from hexadecenal and CTP (C), which in turn catalyzes the formation of CDP-ethanolamine from phosphoethanolamine, were increased in HCC tissues compared with non-tumorous tissues (*P* = 0.011 and *P* <0.001, n = 77). The asterisk indicates a significant difference. (D) The mRNA expression levels of SPL in HCC tissues compared with non-tumorous tissues correlated with the degree of tumor differentiation.

Next, we examined the potential mechanism underlying the reduced levels of S1P in HCC tissues compared with non-tumorous tissues, despite the higher levels of SK mRNAs in HCC tissues. We wondered whether enhanced S1P degradation might play a role in this phenomenon. S1P is known to be degraded by two distinct pathways: (i) dephosphorylation by SPPs to form sphingosine, which may be reused in sphingolipid metabolism, or (ii) irreversible degradation by SPL to yield fatty aldehyde (hexadecenal) and phosphoethanolamine (Fig 1A) [31]. Thus, we measured the mRNA expression levels of SPP1 and SPL in HCC and in non-tumorous tissues. As shown in Fig 3B, SPP1 mRNA expression levels did not differ between HCC and non-tumorous tissues, whereas SPL mRNA levels were elevated in HCC tissues (Fig 3A). It is also possible that active movement of S1P from inside to outside the cells (Fig 1A) through S1P transporters (SPNS2, spinster homolog 2; ABCC1, ATP-binding cassette sub-family C member 1; ABCG2, ATP-binding cassette sub-family G member 2) [32] might reduce S1P levels in HCC tissues. However, no differences were detected between HCC tissues and non-tumorous tissues with respect to SPNS2, ABCC1 or ABCG2 mRNA levels (Fig 3B). In addition, mRNA expressions of S1P receptors in HCC tissues were examined, showing the increase in S1P1 and S1P2 mRNA levels (S1 Fig). These results suggest that the enhanced mRNA levels of SPL may explain the reduced levels of S1P in HCC tissues compared with non-tumorous tissues, despite the higher levels of SK mRNAs.

Evidence for increased mRNA levels of SPL in HCC tissues compared with non-tumorous tissues prompted us to examine the enzyme levels following S1P degradation by SPL (Fig 1A). As shown in Fig 3C, the mRNA levels of fatty aldehyde dehydrogenase (FALDH), which catalyzes the formation of hexadecenoic acid from hexadecenal and phosphoethanolamine cytidyltransferase (CTP), which in turn catalyzes the formation of CDP-ethanolamine from phosphoethanolamine, were increased in HCC tissues compared with non-tumorous tissues. These results suggest that the SPL pathway (Fig 1A) may be more highly activated in HCC tissues than in non-tumorous tissues.

We then examined the feature(s) of HCC associated with high SPL mRNA expression levels. A high SPL mRNA ratio between HCC tissues and non-HCC tissues correlated inversely with the degree of differentiation in HCC (Fig 3D; *P* = 0.043).

### Higher mRNA Levels of SK2 as a Risk for Intra- and Extra-Hepatic Recurrence after Surgical Treatment

**Fig 4:**
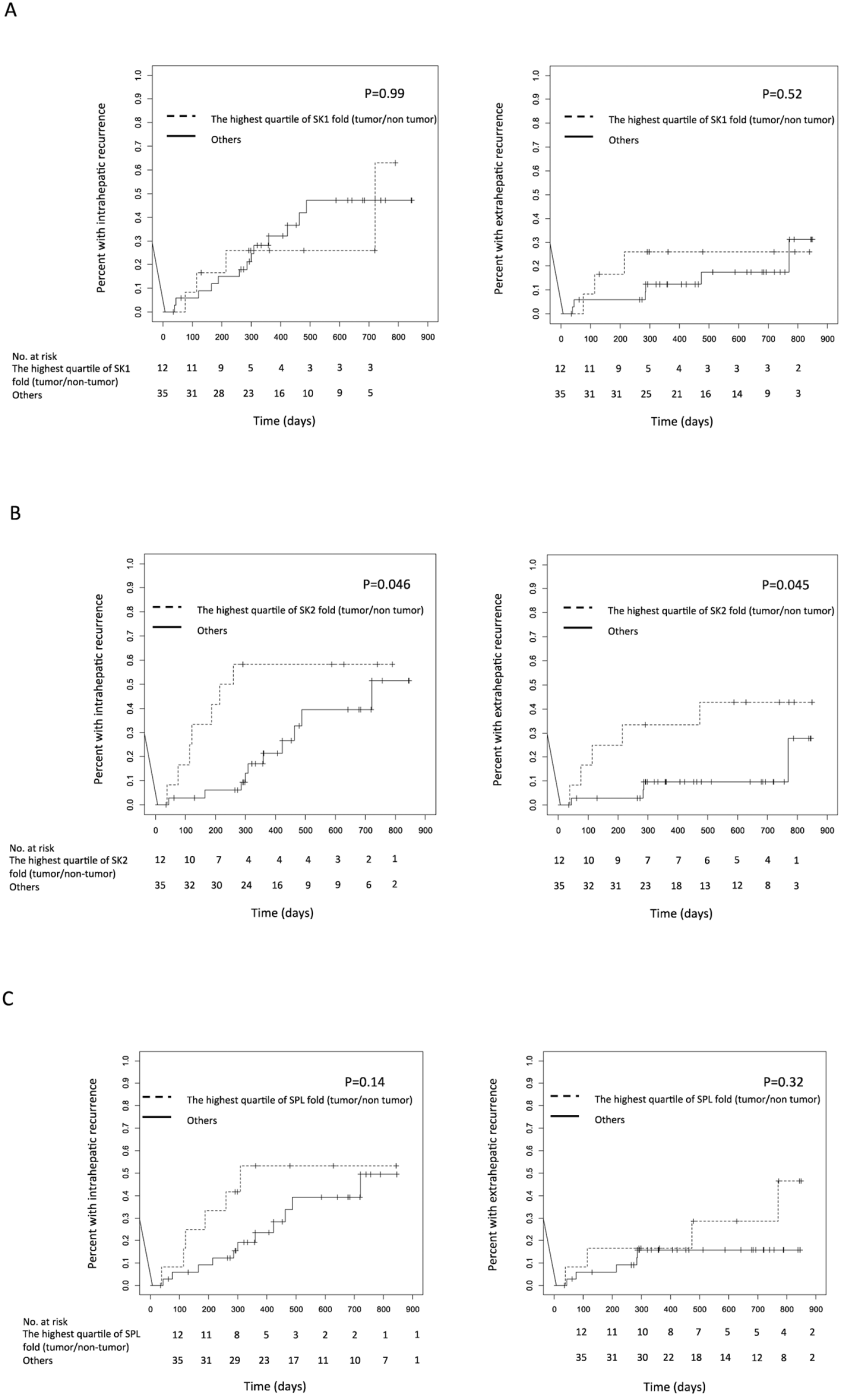
Risk for intra- and extra-hepatic recurrence after surgical treatment. Kaplan-Meier intra- and extra-hepatic recurrence curves were evaluated for patients subdivided according to mRNA levels of SK1 (A), SK2 (B) and SPL (C) in HCC tissues. The dashed line indicates the highest quartile of SK1, SK2 and SPL mRNA ratios between HCC tissues and non-tumorous tissues; the solid line indicates all other patients.

The 47 enrolled patients with primary HCC were followed-up to evaluate HCC recurrence. During the median follow-up period of 358.0 days (1st–3rd quartile: 262.5–609.0 days), 17 patients experienced HCC recurrence, which was analyzed according to the mRNA expression levels of SKs or SPL in the original HCC tissues. The cumulative intra- and extra-hepatic recurrence rates estimated by the Kaplan-Meier method were both higher in the HCC patients, who presented ratios of SK2 mRNA levels (tumor/non-tumor) in the highest quartile compared with the other HCC patients; median disease-free survival was 237 days in patients with the highest quartile of SK2 fold, and 360 days in the other patients. However, the recurrence rates did not differ between the HCC patients, who presented ratios of SK1 mRNA levels (tumor/non-tumor) in the highest quartile compared with the other HCC patients, as demonstrated in Fig 4A and 4B. In contrast, a higher rate of cumulative intra-hepatic recurrence was noted in the HCC patients with SPL mRNA ratios in the highest quartile compared with the other HCC patients, although this difference was not statistically significant (Fig 4C).

### Inhibition of SKs or SPL Expression Led to Reduced Proliferation, Migration and Invasion, while Overexpression of SKs or SPL Caused Enhanced Proliferation of HCC Cells

**Fig 5:**
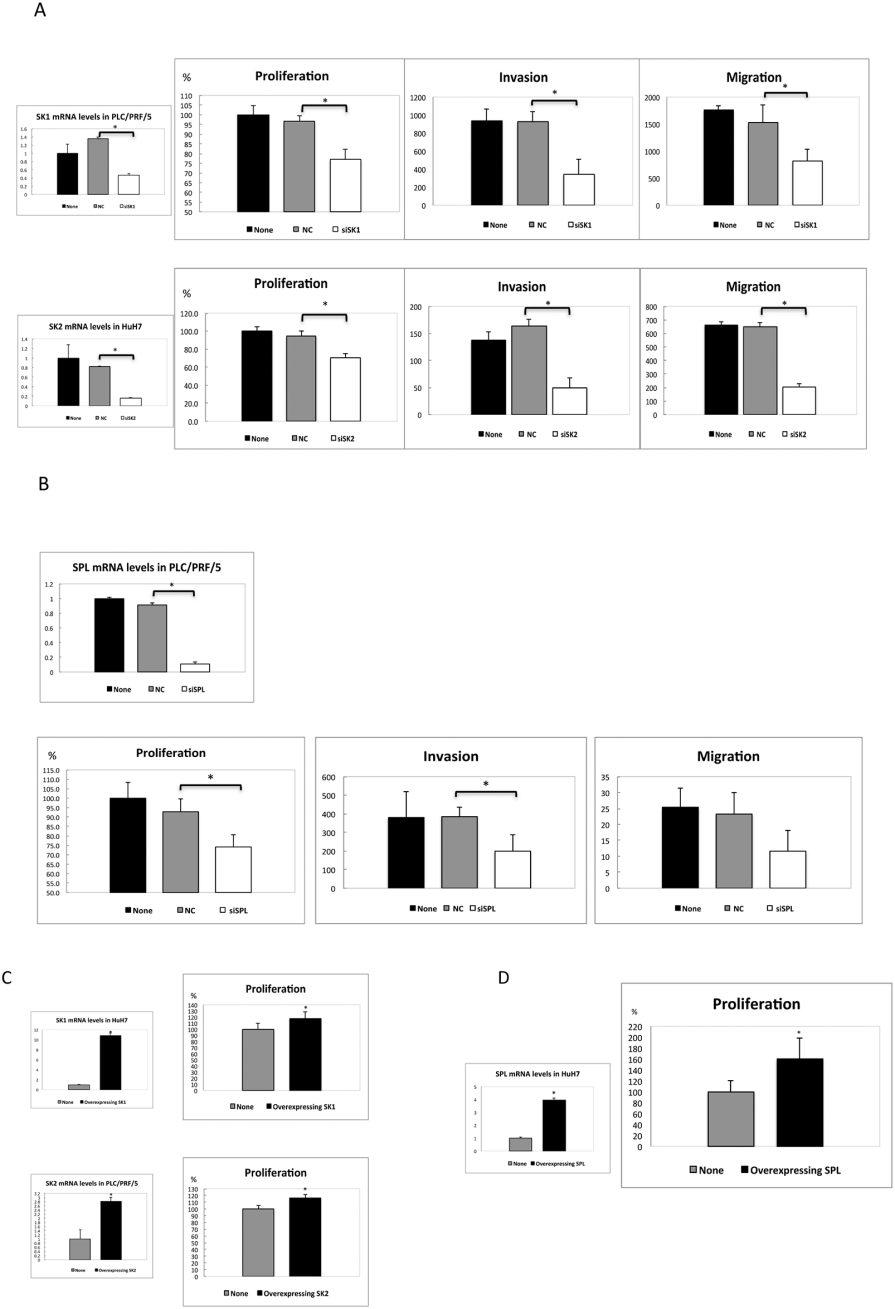
Effects of overexpression and inhibition of SKs or SPL expression. (A, B) PLC/PRF/5 cells transfected with SK1 siRNA and SPL siRNA and HuH7 cells transfected with SK2 siRNA had reduced SK1, SK2 and SPL mRNA levels, respectively, compared with the cells that were transfected with the negative control siRNA (NC; *P* <0.0001, *P* <0.0001and *P* <0.0001, n = 3). Reduced proliferation, invasion and migration were observed as follows: SK1 siRNA *P* <0.001, *P* = 0.007 and 0.03; SK2 siRNA *P* <0.0001, *P* = 0.003 and *P* <0.0001; SPL siRNA *P* = 0.0002, *P* = 0.03 and *P* = 0.09, n = 3. (C, D) The FLAG-tagged mammalian expression vector p3FLAG-CMV10 enabled us to obtain stably transfected cells expressing SK1 (HuH7 cells), SK2 (PLC/PRF/5 cells) and SPL (HuH7 cells) (*P* <0.0001, 0.0001 and 0.0001, n = 3). In these cells, proliferation was enhanced compared with the non-transfected cells (*P* <0.0001, *P* = 0.0002, and *P* <0.0001, n = 3). The asterisk indicates a significant difference.

To gain insight regarding the significance of the high mRNA levels of SKs or SPL in HCC, we examined the potential effect of inhibiting the expression of SKs or SPL in HCC cell lines. We employed PLC/PRF/5 cells for SK1 and SPL inhibition, whereas HuH7 cells were used for SK2 inhibition because higher mRNA levels of these genes were observed in those HCC cell lines (S2 Fig). As shown in Fig 5A and 5B, our siRNAs for SK1, SK2 and SPL successfully reduced the mRNA levels of SK1, SK2 and SPL compared with the negative control siRNAs. In addition, reduced proliferation, invasion and migration were observed in PLC/PRF/5 cells transfected with SK1 siRNA (Fig 5A) and in HuH7 cells transfected with SK2 siRNA (Fig 5A) compared with the cells that were transfected with the negative control siRNA. This finding suggests that SK1 and SK2 may play a role in the proliferation, invasion and migration of HCC cells. In contrast, proliferation, invasion and migration were reduced in PLC/PRF/5 cells transfected with SPL siRNA (Fig 5B) compared with the cells that were transfected with negative control siRNA. This result suggests that SPL may also play a role in proliferation, invasion and migration of HCC cells.

Furthermore, we examined the potential effect of overexpression of SK1, SK2 and SPL in HCC cells. To achieve this goal, HuH7 cells were used to overexpress SK1 and SPL, and PLC/PRF/5 cells were used to overexpress SK2 due to the lower mRNA levels of each of these genes in those cells (data not shown). We used the FLAG-tagged mammalian expression vector p3FLAG-CMV10, which enabled us to obtain stably transfected cells expressing SK1, SK2 and SPL (Fig 5C and 5D). In these cells, proliferation was enhanced compared with the non-transfected cells (Fig 5B and 5D), confirming that SK1, SK2 and SPL may play a role in the proliferation of HCC cells. Of note, SPL-transfected cells in culture medium with glucose did not differ in proliferation from none-transfected cells, suggesting that the products of SPL pathway may contribute to proliferation of HCC cells with few other energy sources. Nonetheless, overexpression of SK1, SK2 or SPL may make the cells proliferative to various degrees.

## Discussion

The results of the current study showed that the levels of SK1 and SK2 mRNA were increased in human HCC tissues compared with adjacent non-tumorous tissues in patients who underwent surgery. The mRNA levels of both SK1 and SK2 correlated with poorer differentiation in HCC and microvascular invasion in HCC tissues. Increased mRNA expression of SK2 was further found to be a risk factor for intra- and extra-hepatic recurrence. Thus, our results suggest that SKs play a role in the pathophysiology of HCC. Our current results are consistent with those of previous *in vitro* studies showing that SK1 promotes HCC cell migration and invasion [33] and that an SK2 inhibitor exerts anti-tumor activity in HCC cell lines [34].

Increased SK1 mRNA and/or protein expression has been reported in many cancers other than HCC [12]. This increase in SK1 has been assumed to play an important role in cancer pathophysiology [12] because (i) SK1 generates S1P and (ii) the increased intracellular S1P levels have been shown to be pro-proliferative and anti-apoptotic [2,35]. However, actual S1P levels in human cancer tissues have rarely been measured; only one publication has reported increased S1P levels in human glioblastoma tissues [36], whereas reduced S1P levels have been reported in metastatic pancreatic cancer compared with normal tissues [37]. Thus, additional studies are needed to verify that high SK1 expression in tumors translates to elevated S1P levels in tissues [38]. In this context, our study has revealed for the first time, and to the best of our knowledge, that S1P levels were even lower in human HCC tissues than in adjacent non-tumorous tissues in spite of the increased expression of SK1 and SK2 mRNA.

To address the mechanism underlying the low levels of S1P despite the increased levels of SK1 and SK2 mRNA in HCC tissues, enhanced degradation of S1P was examined. Indeed, among the enzymes that degrade S1P, the expression of SPL mRNA but not SPP1 mRNA was increased in HCC tissues compared with non-tumorous tissues. Furthermore, the mRNA expression levels of the two distinct enzymes that catalyze the formation of S1P metabolites following degradation by SPL were elevated in HCC tissues compared with non-tumorous tissues. These results strongly suggest that the SPL pathway is active in HCC tissues. The increased expression of SPL mRNA in HCC tissues correlated with poorer HCC differentiation, suggesting that SPL may also play a role in the pathophysiology of HCC. Furthermore, our *in vitro* study showed that inhibition of SPL expression by siRNA led to reduced proliferation and invasion, while overexpression of SPL caused enhanced proliferation of HCC cell lines. This finding is in direct contrast to data showing that SPL inhibits tumor cell proliferation and survival of colon cancer cells [39]. The role of SPL in tumor cell proliferation may differ in various cell and cancer types (e.g., colon cancer vs. HCC). In this context, the evidence supports a stimulatory role of SPL in mitogenesis, in which mouse embryonic carcinoma cells with various expression levels of SK1 and SPL were analyzed. Interestingly, overexpression of SPL and SK1 reportedly resulted in the most striking mitogenic effect compared with overexpression of SK1 only or low levels of SPL [40]. It has also been suggested that intracellular S1P plays an important role as an intermediate in the sole sphingolipid-to-glycerophospholipid metabolic pathway [41]. In cancer cells, SPL expression has been shown to be down-regulated in human prostate cancer cells [42] but up-regulated in ovarian cancer cells [43], which suggests that SPL expression levels may also depend on the cancer cell type.

Accumulating evidence has supported an important role for SK1 in cancer cell proliferation or survival. In contrast, recently developed, selective SK1 inhibitors [44,45] and, similarly, novel SK1/2 inhibitors [38,46] had no effect on cancer cell proliferation or survival, although the reason why these SK1 or dual SK1/2 inhibitors did not exert an anti-tumor effect remains to be elucidated. These lines of evidence suggest that the controversy exists regarding roles of SK1/2 in cancer cell proliferation or survival. In this context, our current evidence suggests that the concurrent application of an SPL inhibitor with SK1 or dual SK1/2 inhibitors may inhibit cancer cell proliferation or survival, depending on the cell types. Thus, a role for intracellular S1P in cancer cell proliferation or survival should also be considered.

In conclusion, higher levels of SK and SPL mRNA and lower levels of S1P in human HCC tissues compared with adjacent non-tumorous tissues were observed for the first time in the present study. These increased levels of SK and SPL mRNA in HCC tissues correlated with poorer HCC differentiation. The activation of both SKs and SPL may play a role in HCC cell proliferation, migration and invasion, suggesting that both SKs and SPL may be potential targets for the treatment of HCC.

## Supporting Information

S1 Fig S1P receptors mRNA expression levels. mRNA expressions of S1P receptors in HCC tissues were showing the increase in S1P1 and S1P2 mRNA levels. The asterisk indicates a significant difference.
(PPTX)

S2 Fig Expression levels of SK1, Sk2 and SPL mRNA in HCC cell lines. PLC/PRF/5 cells were expressing high levels of SK1 and SPL, whereas HuH7 cells were expressed high levels of SK2.
(PPTX)

S1 Table LC-MS/MS conditions for sphingolipid analysis. (PDF)

S2 Table List of primers used for S1PRs mRNA measurement. (PPTX)
