# Supplementary figures and images for: Increased mRNA Levels of Sphingosine Kinases and S1P Lyase and Reduced Levels of S1P Were Observed in Hepatocellular Carcinoma in Association with Poorer Differentiation and Earlier Recurrence
Source: PLoS One. 2016 Feb 17;11(2):e0149462. doi: 10.1371/journal.pone.0149462 (PMC4757388; doi:10.1371/journal.pone.0149462)

## Slide 1
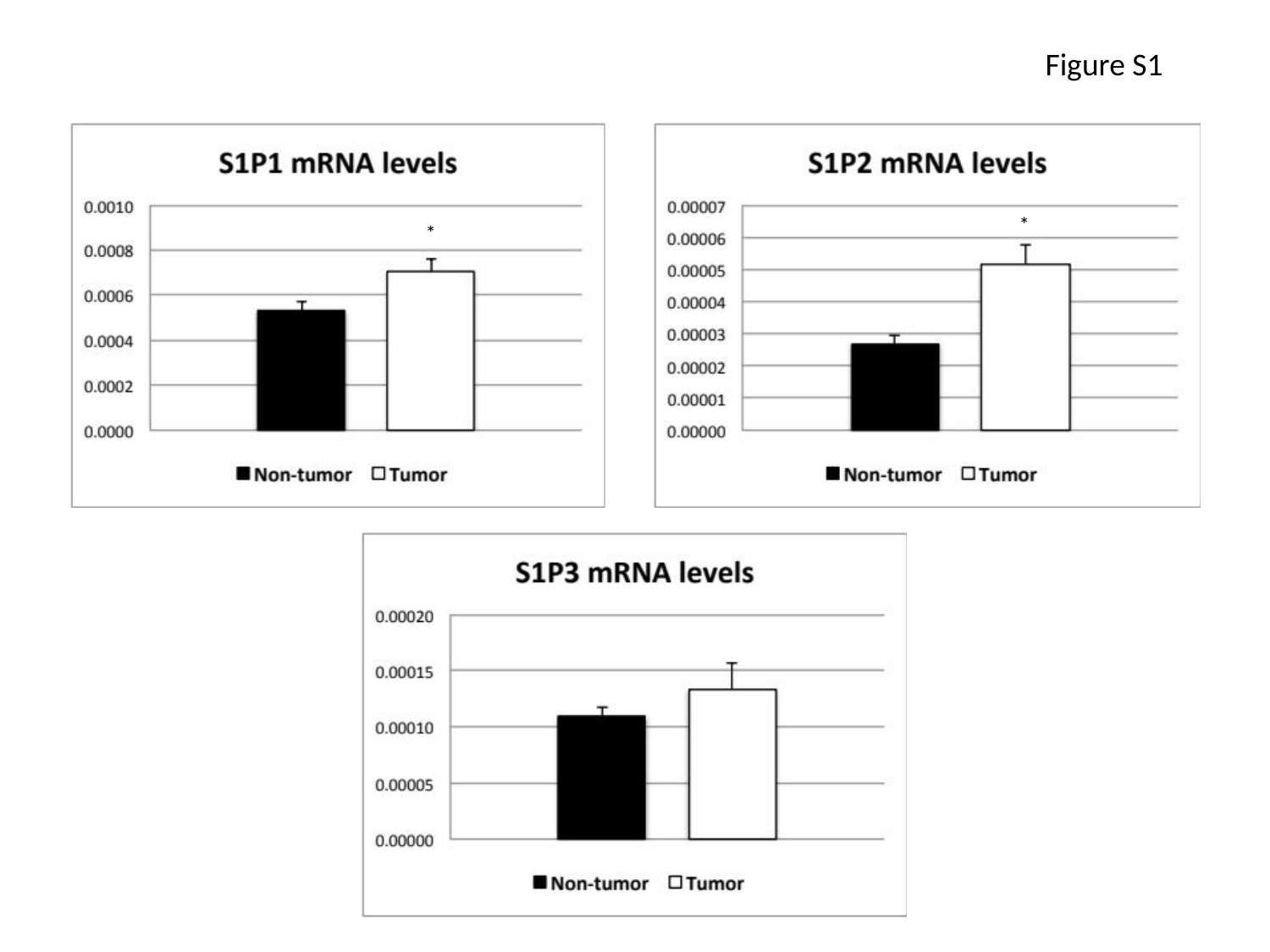

Figure S1
*
*

Supplement: S1 Fig — mRNA expressions of S1P receptors in HCC tissues were showing the increase in S1P1 and S1P2 mRNA levels. The asterisk indicates a significant difference. (PPTX) [file pone.0149462.s001.pptx]

## Slide 1
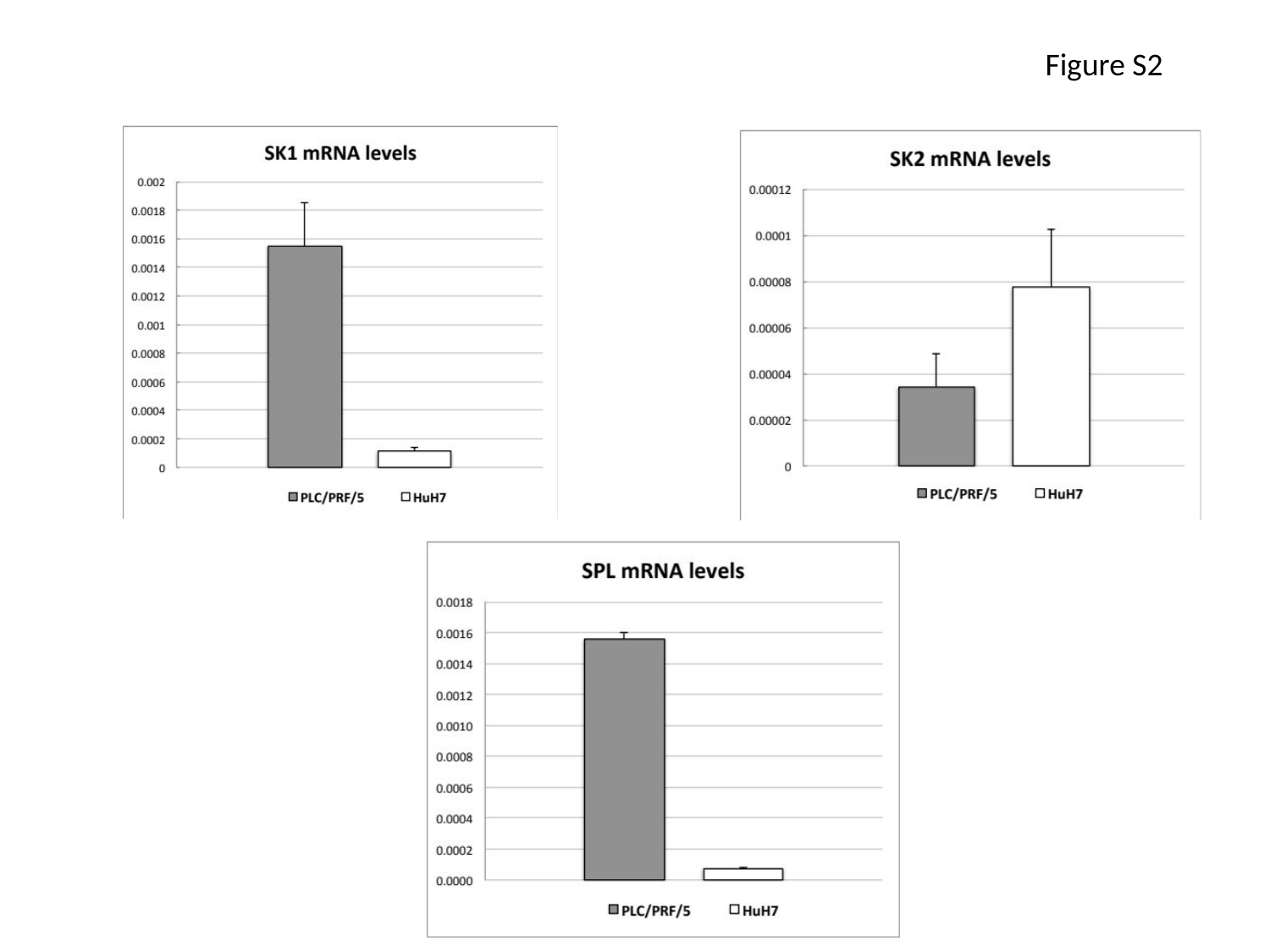

Figure S2

Supplement: S2 Fig — PLC/PRF/5 cells were expressing high levels of SK1 and SPL, whereas HuH7 cells were expressed high levels of SK2. (PPTX) [file pone.0149462.s002.pptx]
