# Supplementary material for: Increased mRNA Levels of Sphingosine Kinases and S1P Lyase and Reduced Levels of S1P Were Observed in Hepatocellular Carcinoma in Association with Poorer Differentiation and Earlier Recurrence
Source: PLoS One. 2016 Feb 17;11(2):e0149462. doi: 10.1371/journal.pone.0149462 (PMC4757388; doi:10.1371/journal.pone.0149462)
Supplement: S1 Table — (PDF) [file pone.0149462.s003.pdf]

Table S1. LC-MS/MS condition for sphingolipids analysis

MS condition	
Ionization	ESI (+)
Spray voltage	3,000V
Vaporizer temperature	450 °C
Capillary temperature	350 °C
Sheath gas pressure	0.34 Mpa
Auxiliary gas pressure	0.14 Mpa
Collision gas pressure	2.0 mTorr

SRM condition	Precursor ion (*m/z*)	Product ion (*m/z*)	Collision energy (eV)
Sph	300.3	282.2	11
S1P	380.3	264.2	16
Cer d18:1/16:0	538.7	264.2	23
Cer d18:1/22:0	622.7	264.2	27
Cer d18:1/24:1	648.7	264.2	27

HPLC condition	
Analytical column	Capcell Pak C8 UG120 (100 mm × 1.5 mm i.d., 5 µm particle size) (Shiseido)
Mobile phase	Gradient<br>A: 5 mmol L^{-1} ammonium formate in water, pH 4.0<br>B: 5 mmol L^{-1} ammonium formate in 95% (v/v) acetonitrile, pH 4.0<br>Initial: A/B=70/30<br>0-1.0 min: 70/30, 1.0-8.0 min: 70/30→0/100, 8.0-23.0 min: 0/100,<br>23.0-23.1 min:0/100→70/30
Flow rate	300 µL/min
Oven temperature	40 °C
