# Supplementary material for: Increased mRNA Levels of Sphingosine Kinases and S1P Lyase and Reduced Levels of S1P Were Observed in Hepatocellular Carcinoma in Association with Poorer Differentiation and Earlier Recurrence
Source: PLoS One. 2016 Feb 17;11(2):e0149462. doi: 10.1371/journal.pone.0149462 (PMC4757388; doi:10.1371/journal.pone.0149462)
Supplement: S2 Table — (PPTX) [file pone.0149462.s004.pptx]

## Slide 1
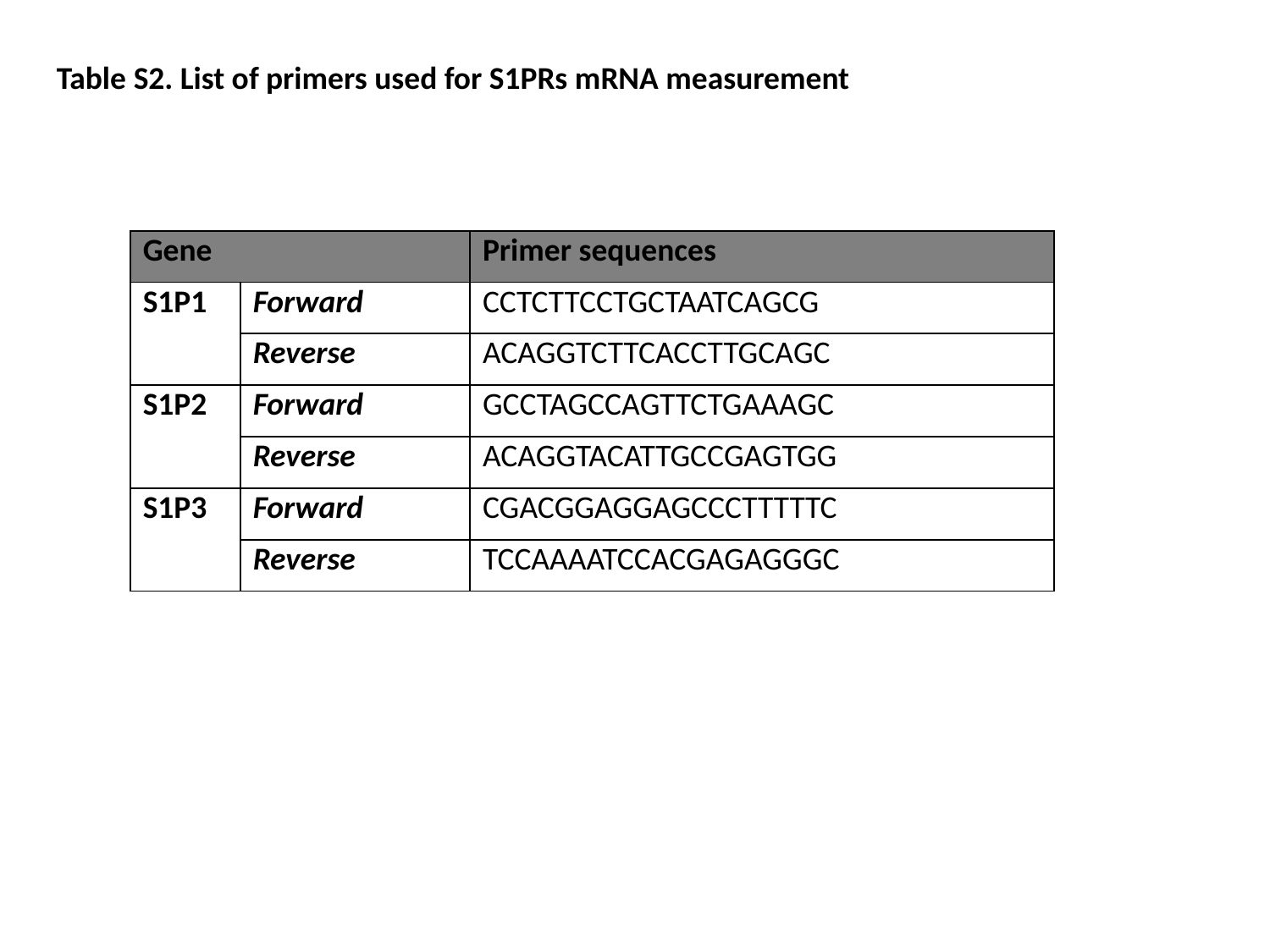

Table S2. List of primers used for S1PRs mRNA measurement
| Gene | | Primer sequences |
| --- | --- | --- |
| S1P1 | Forward | CCTCTTCCTGCTAATCAGCG |
| | Reverse | ACAGGTCTTCACCTTGCAGC |
| S1P2 | Forward | GCCTAGCCAGTTCTGAAAGC |
| | Reverse | ACAGGTACATTGCCGAGTGG |
| S1P3 | Forward | CGACGGAGGAGCCCTTTTTC |
| | Reverse | TCCAAAATCCACGAGAGGGC |
